# Flex-sweep 2.0: more flexible and faster selective sweeps detection

**DOI:** 10.64898/2026.08.06.743046

**Published:** 2026-08-06

**Authors:** Jesús Murga-Moreno, David Enard

## Abstract

Flex-sweep is a convolutional neural network-based method able to detect a wide range of selective sweeps, including those thousands of generations old, from single population genomic data, while robust to background selection. Here we present a substantial update that streamlines the entire workflow. The new version vastly reduces memory needs and vastly speeds up summary-statistic computation over fully customizable statistics combinations and genomic regions, relaxes CNN constraints by supporting custom architectures and haplotype matrix sorting methods. Domain-Adaptive Neural Network (DANN) training is now supported, as well as ancestral-state polarization and a robust, clustering and confounder-aware gene set sweep enrichment pipeline robust for downstream analysis. Flex-sweep 2.0 scales to hundreds of thousands of training simulations, and enables genome-wide inference on a standard workstation.

## Full Text Availability

The license terms selected by the author(s) for this preprint version do not permit archiving in PMC. The full text is available from the preprint server.

